# Comparison of two reinforcement rings for primary total hip arthroplasty addressing displaced acetabular fractures: a biomechanical analysis

**DOI:** 10.1007/s00402-020-03433-3

**Published:** 2020-04-08

**Authors:** Johannes Becker, M. Winkler, C. von Rüden, E. Bliven, P. Augat, H. Resch

**Affiliations:** 1 Department of Trauma Surgery, BG Unfallklinik Murnau, Murnau, Germany; 2 Institute for Biomechanics, BG Unfallklinik Murnau, Murnau, Germany; 3 Institute for Biomechanics, Paracelsus Medical University, Salzburg, Austria; 4 Department of Traumatology and Sports Medicine, Paracelsus Medical University, Salzburg, Austria

**Keywords:** Displaced acetabular fracture, Hip arthroplasty, Acetabulum roof reinforcement plate, Burch–Schneider reinforcement cage

## Abstract

**Introduction:**

Aim of this study was to biomechanically compare two different acetabular cup fixation constructs in terms of fracture fixation for displaced acetabular fractures involving the anterior column with hemitransverse fracture under partial and full weight-bearing conditions.

**Methods:**

Two different reinforcement rings designed as cages for primary THA were biomechanically tested in terms of managing a complex acetabular fracture. Single-leg stance cyclic loading was performed to assess fracture gap movement and fragment rotation. Twelve hemi pelvis Sawbones were divided into two groups: primary THA with acetabulum roof reinforcement plate (ARRP) (*n* = 6) and primary THA with Burch–Schneider reinforcement cage (BSRC) (*n* = 6).

**Results:**

During loading under partial weight-bearing (250 N) fracture gap movement tended to be larger in the BSRC group as compared to the ARRP group. Under full weight-bearing conditions, the ARRP showed 60% significantly less motion (*p* = 0.035) of the *os ilium* to *os ischii* gap compared to BSRC. Fracture gap movements between the *os ilium* and *spina iliaca* fragments were significantly reduced by 76% (*p* = 0.048) for ARRP in contrast to BSRC. The ARRP group also demonstrated significantly less movement in the fracture gaps *os ischii* to *quadrilateral plate* (62% reduction, *p* = 0.009) and *quadrilateral plate* to *spina iliaca* (87% reduction, *p* < 0.001). Significantly less rotational movement of the *quadrilateral plate* to the *os ilium* was exhibited by the ARRP group (*p* = 0.015).

**Conclusions:**

The presented acetabulum roof-reinforcement plate (ARRP) provides stable conditions at the acetabular component with adequate stabilization of a displaced acetabular fracture.

## Introduction

The incidence of anterior column fractures combined with hemitransverse (ACPHT) fractures tremendously increases due to an aging society. Such fractures involve displacement of the quadrilateral plate (QLP) and are often associated with a higher degree of comminution and impaction in patients with osteoporotic bone quality [15, 23, 25, 42]. Stable fixation and anatomical reduction of this “key” fragment is mandatory, but also very challenging due to reduced bone quality [23, 27, 42]. Primary total hip arthroplasty (THA) and the use of cages for joint reconstruction offer the advantage of stable fixation and the possibility of immediate postoperative mobilization with full weight-bearing [4, 26, 29–31, 34, 43].

In the past, only a few biomechanical studies have analyzed the stability of acetabular fracture reconstruction methods underlining the necessity of stable osteosynthesis to prevent re-displacement of the QLP [6, 24]. However, the lack of homogeneity in biomechanical test set-ups aggravates comparison under full weight-bearing conditions [6, 7, 10, 17, 22, 24, 33, 36]. In particular no comparative biomechanical data has been made available regarding cages designed for primary THA which address displaced acetabular fractures in the elderly.

This study compares two different reinforcement cages for displaced acetabular fractures providing fixation of the acetabular roof. Designed as a defect implant for revision surgery in hip bone defects, the Burch–Schneider reinforcement cage (BSRC) aims to restore the anatomical rotational center of the hip [40]. The fixation of the BSRC is achieved by trabecular screws which may become challenging in the case of multi-fragmental fractures due to reduced options for screw placement. On the other hand, the newly designed acetabulum roof-reinforcement plate (ARRP) is intended as a fracture fixation cage and offers multiple options for the insertion of fixed-angle stable screws [29, 30]. We hypothesize that the acetabulum roof-reinforcement plate offers higher biomechanical stability in comparison to the Burch–Schneider reinforcement cage in terms of prevention of quadrilateral plate protrusion and fracture gap movements under partial and full weight-bearing conditions.

## Materials and methods

### Specimens and preparation

**Fig. 1:**
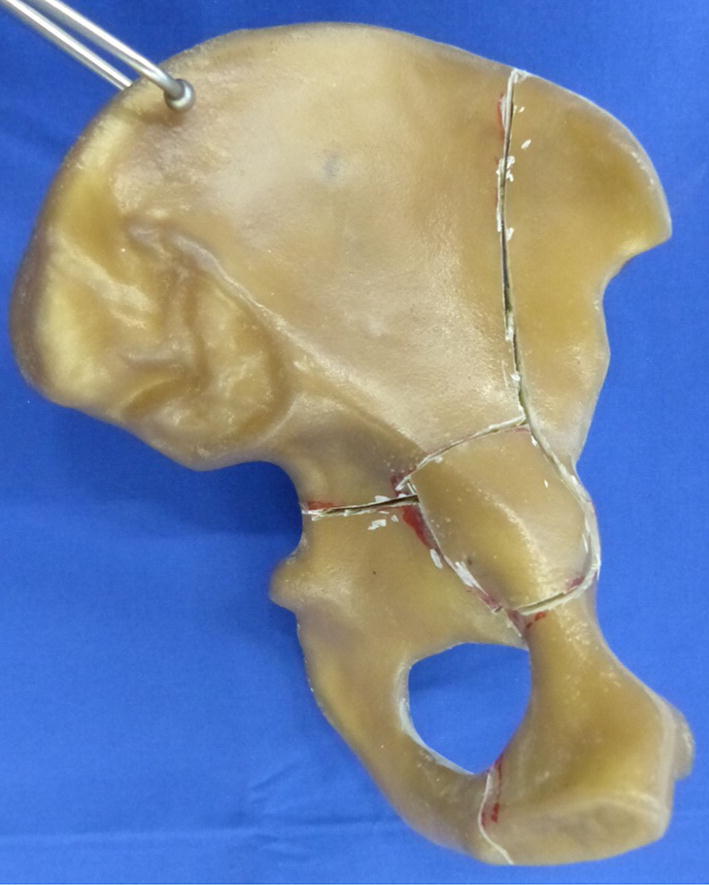
Fracture of the anterior column combined with a posterior hemi transverse fracture and additional break out of the quadrilateral plate. Fracture lines were achieved using an oscillating saw and a 3D-printed template

The study was conducted using synthetic hemi pelvises (3405 left pelvis-partial, 4th Generation, Sawbones, Malmö, Sweden). A CT scan of one synthetic hemi pelvis bone was performed and DICOM data of the scan was segmented. After data segmentation, a negative of the prepared hemi pelvis model was used to create a virtual sawing template. This template was 3D printed with PolyJet technology. Using this template, an anterior column combined with posterior hemitransverse (ACPHT) fracture with additional break out of the quadrilateral plate (Fig. 1) was reproducibly cut in twelve synthetic bones with an oscillating saw, as according to Culemann et al. [6]. Two groups of six hemi pelvis Sawbones each were created and prepared for cyclic loading: ARRP, primary THA with Acetabulum Roof Reinforcement Plate (41medical AG, Bettlach, Switzerland) and BSRC, primary THA with Burch-Schneider Reinforcement Cage (Zimmer Biomet Deutschland GmbH, Freiburg i. Breisgau, Germany).

Prior to testing, specimens were attached to an artificial sacrum that consisted of a polyurethane cast (RenCast FC 53 A/B, Gößl + Pfaff GmbH, Karlskron/Brautlach, Germany) which was used to create an equal load distribution between the servohydraulic testing machine and synthetic bone, mimicking the sacroiliac joint. For correct placing of the sacrum substitute, anatomical correlations and geometric dimensions of a fourth generation Sawbones sacrum (3405, Sawbones, Malmö, Sweden) corresponding to the hemi pelvis were used for manufacturing. The connection between sacrum and hemi pelvis was secured using three threaded rods (M8) with corresponding nuts. The artificial sacrum was reused for each test sample.

### Implant configurations

**Fig. 2:**
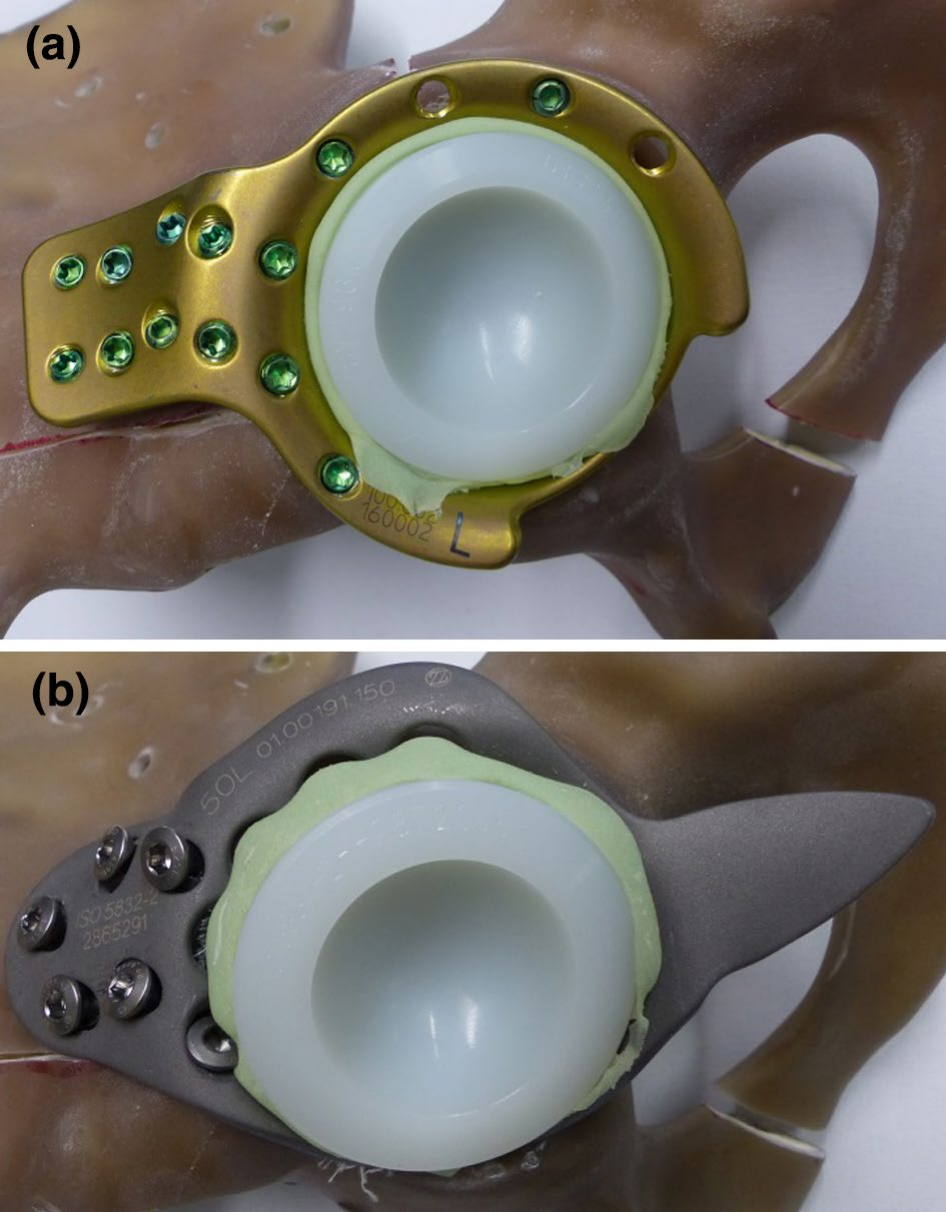
Specimens were randomized into two different groups. Construct A is an acetabulum roof reinforcement plate (ARRP), locked with 13 3.5-mm-diameter locking screws. Construct B is a Burch–Schneider reinforcement cage (BSRC) secured with seven 6.5-mm-diameter cancellous screws. One screw of construct B is located below the cementation

The bones were randomly assigned to one of two methods of primary THA (Fig. 2): ARRP implants were fixated in the acetabulum with 13 3.5 mm titanium self-tapping locking screws (Johnson & Johnson Medical GmbH, DePuy Synthes, Umkirch, Germany). BSRC implants were fixated with seven 6.5 mm Countersunk Cancellous Bone Screws (Protasul™-100, Zimmer Biomet Deutschland GmbH, Freiburg i. Breisgau, Germany). Screw lengths for both constructs were chosen to secure the implants bicortically. Polyethylene inlays (Sulene™-PE, Zimmer Biomet Deutschland GmbH, Freiburg i. Breisgau, Germany) with a 50 mm outer and a 32 mm inner diameter were cemented in all implants (Palacos R, Heraeus Medical GmbH, Wehrheim, Germany). Implantation and cementation of all constructs was performed by one experienced surgeon according to the manufacturer’s recommendations.

### Test setup

**Fig. 3:**
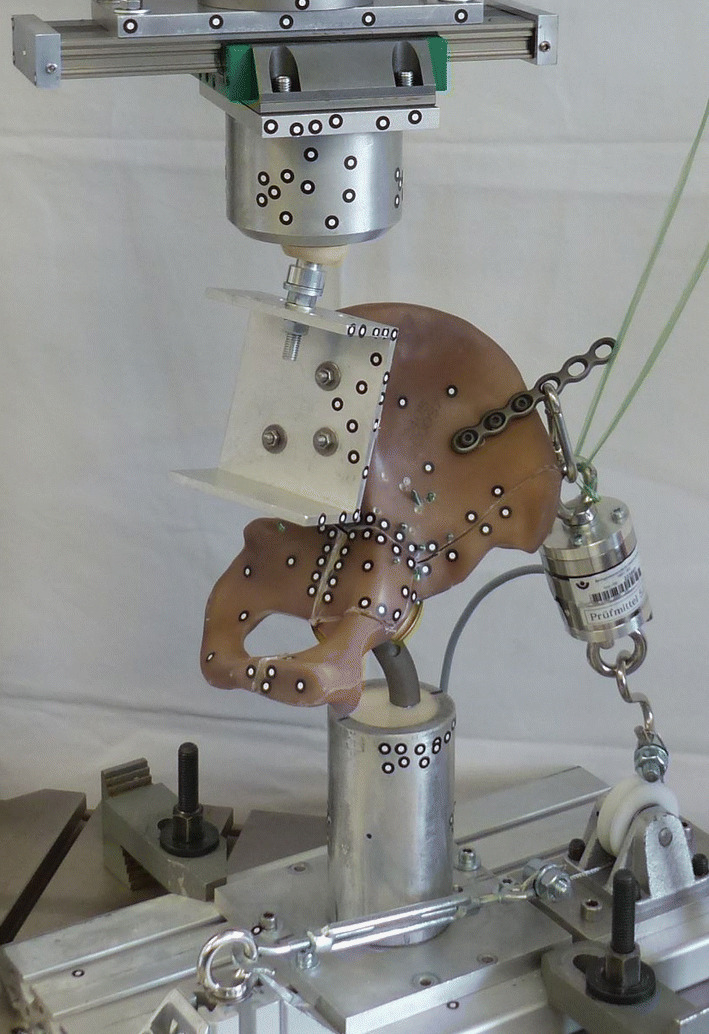
Biomechanical test set-up. Axial compression tests were performed on all specimens to simulate one-legged stance. To stabilize the test constructs, additional tension was applied over a plate fixated at the *os ilium*. Traction was measured with a secondary load cell

Biomechanical testing was conducted using an Instron 8874 servohydraulic testing machine (Instron Deutschland GmbH, Darmstadt, Germany). A single-leg stance model (Fig. 3) was created in accordance with previous studies [28, 38, 41]. Force was applied proximally at the sacrum substitute via an artificial acetabular cup embedded in an aluminum cylinder. The cylinder was connected to the actuator of the testing machine, which included a biaxial load cell (Instron Dynacell, measuring range ± 10 kN, ± 100 Nm, Instron Deutschland GmbH, Darmstadt, Germany). All specimens were connected via the sacrum surrogate using a 36 mm diameter ceramic ball mounted on a U-beam. The U-beam was fixed to a linear slide ensuring a vertical force direction by enabling frontal movement. It was attached to the load cell and the aluminum cylinder to avoid undesirable constraint forces. The limited sagittal movement of the slide was balanced due to the ceramic ball mounted in the aluminum cylinder applying loads during biomechanical testing and allowing movement of the test specimen in all three axes. Distally, a revision stem (SL Revision Stem 17 L 265, Sulzer Orthopedics Ltd., Baar, Switzerland) was embedded in a cylindrical aluminum pod. The polyurethane resin described above was used as embedding material. A 32 mm diameter femoral head connected test constructs with the stem. Additionally, a wire was mounted onto a plate directly attached to the ala of ilium caudally to the iliac crest and dorsal to the anterior superior iliac spine to prevent the pelvis from collapsing. Tension in the cable was measured with a load cell (U3, measuring range 1 kN, HBM GmbH, Darmstadt, Germany) and a turnbuckle was used to achieve correct hip contact force angles by changing the cable length. Due to confined space, a pulley was used to switch wire direction.

### Test procedure

**Fig. 4:**
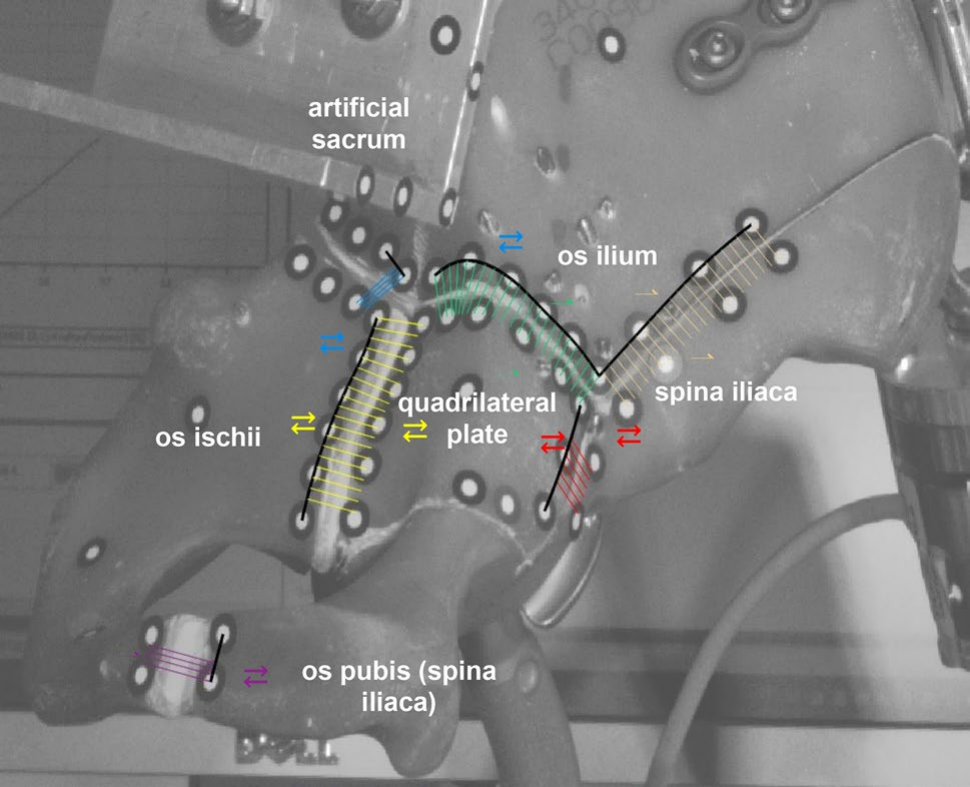
Fracture lines and bone fragments of the hemi pelvis. Marker points were placed along fracture lines and captured with an optical measurement system during testing. *Spina iliaca* and *os pubis* represent one connected fragment, which was differentiated for a better anatomical description

In-vivo hip contact force angles resulting during single leg stance were obtained as according to Bergmann et al. [3]. Loading angles from this data were set on each construct prior to testing by adjusting cable length with the turnbuckle, causing test constructs to tilt to the right position. When constructs were loaded axially with the testing machine’s actuator, a secondary load occurred due to the additional cable used to prevent the bones from collapsing. This secondary load rose along with an increasing axial load. Therefore, the effective load in the acetabulum was the sum of the primary and secondary loads. This summation was validated using a six degrees of freedom load cell (K6D68, measuring range 5 kN, 50 Nm, ME-Meßsysteme GmbH, Hennigsdorf, Germany) in preliminary tests. In the actual tests samples underwent a cyclic test protocol with increasing load. Testing was performed until the upper load limit was reached. Axial load was applied via the ball joint on the artificial sacrum as described above. The protocol started with a ramp to a 50 N lower level set point, then followed by cyclic sinusoidal fatigue loading with an initial 250 N upper axial primary load, increasing by 50 N every 1000 cycles to a maximum of 1800 N, resulting in a total of 31,000 cycles. The secondary passive load was in the range of 400–600 N at the end of loading, which resulted in an overall maximum load level of 2200–2400 N. These loads were chosen to simulate the largest resulting hip loads during single leg stance (1753–2505 N), as according to data from Bergmann et al. [3]. A 250 N initial upper load was selected to mimic partial weight-bearing, common after hip surgery in a patient with average body weight. Movements of the fracture gaps, i.e., opening and closing, were measured and calculated using a three dimensional motion analysis video system (ARAMIS 5 M, GOM GmbH, Braunschweig, Germany), which tracked point markers placed along the fracture lines (Fig. 4). Gap opening and closing were evaluated by calculating the overall relative movement within all three axes.

### Data analysis

Fracture gap movements and quadrilateral plate fragment rotation with respect to the *os ilium* were determined and statistically analyzed (IBM SPSS Statistics 19, Chicago, IL, USA). For statistical analysis, a Student’s *t* test for independent samples was performed to identify differences between the two groups. Distribution for normality was checked with Shapiron–Wilk test. Results are presented as mean value ± standard deviation (SD).

## Results

**Fig. 5:**
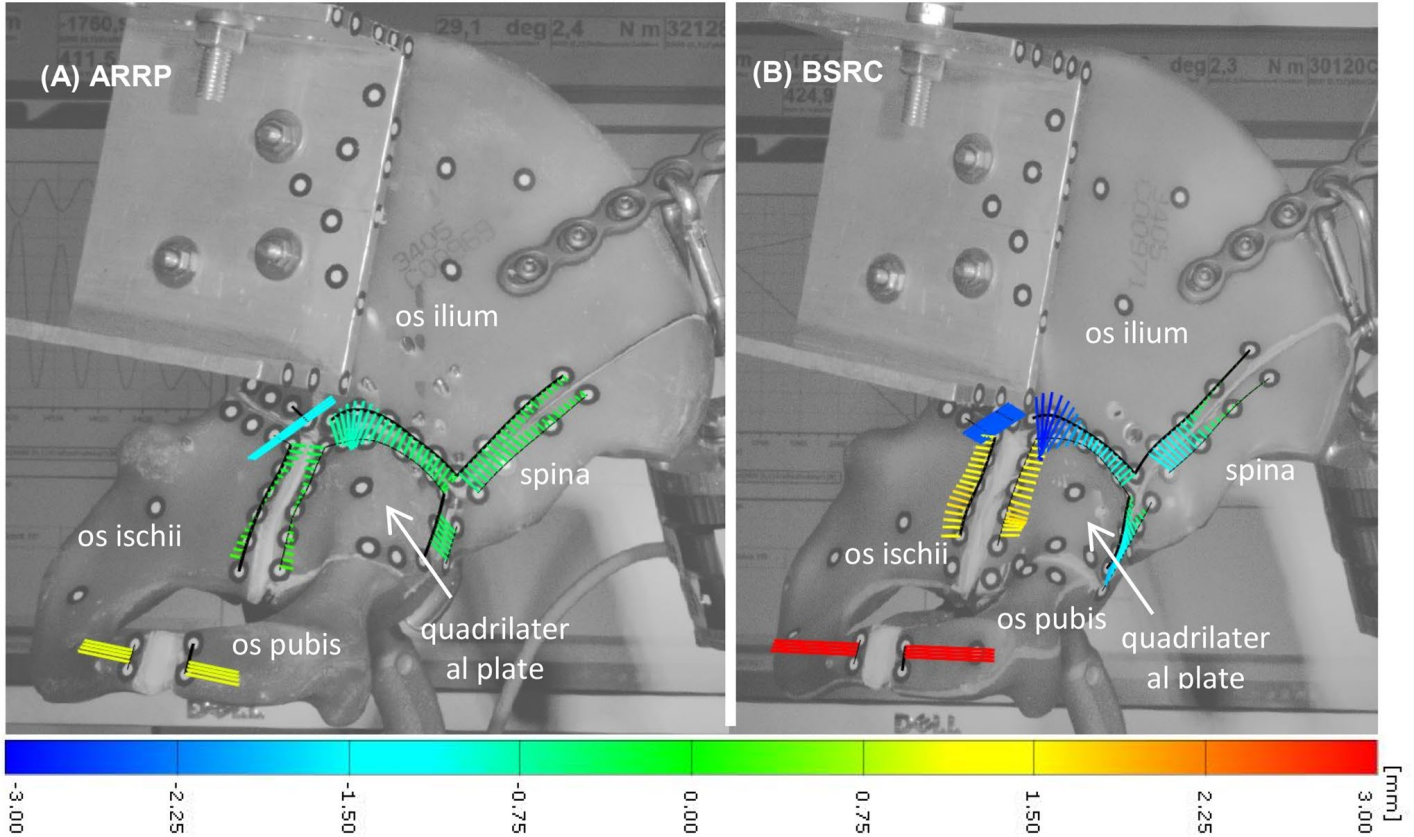
Fracture gap movements of one characteristic specimen from each group, **a** ARRP and **b** BSRC, after 31,000 cycles under full weight bearing. Colored lines indicate the fracture gap movements in mm. Colors approaching red show divergence, and colors approaching blue convergence

**Fig. 6:**
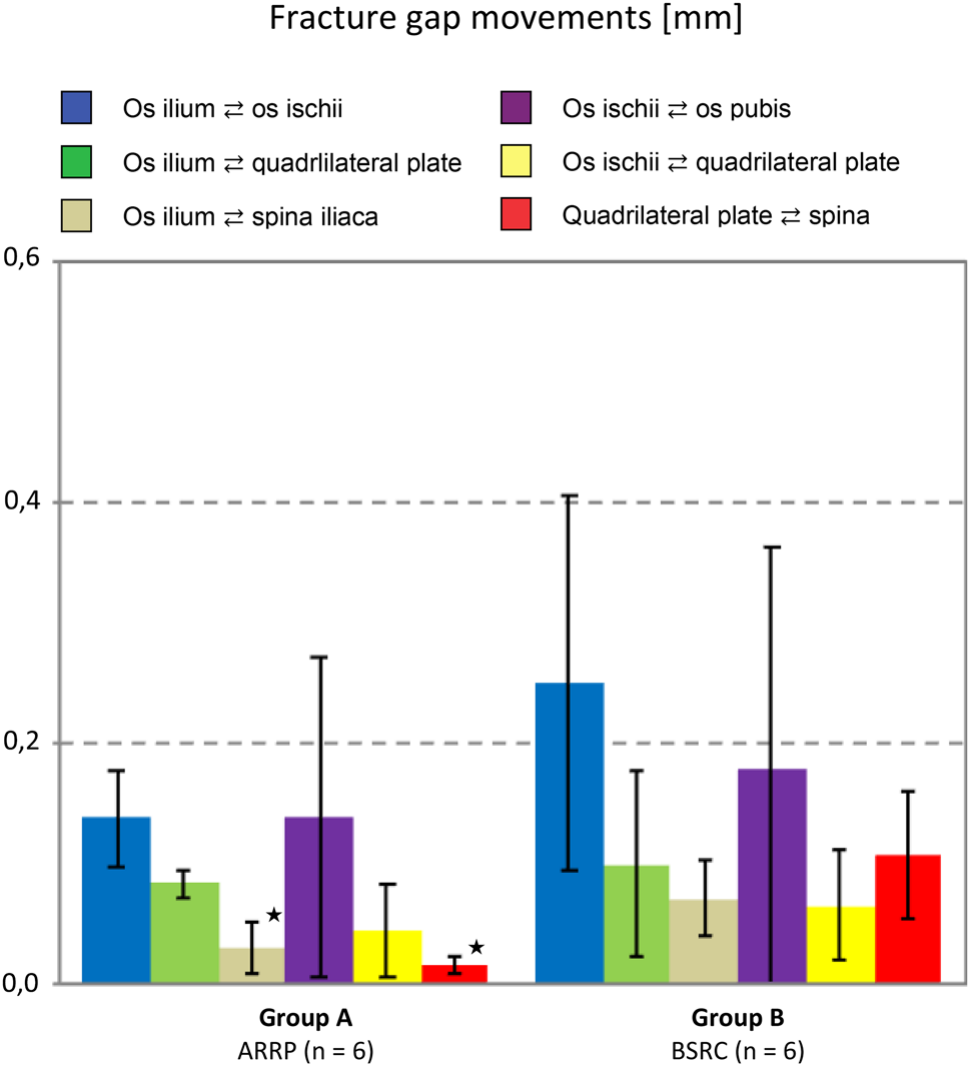
Fracture gap movements for the tested groups A and B (mean value ± 0.1 SD) after 1000 cycles under partial weight-bearing. Student’s *t* tests for independent samples were performed to identify differences in the same fracture gaps among the two groups. Stars indicate statistically significant (*p* < 0.05) results

**Fig. 7:**
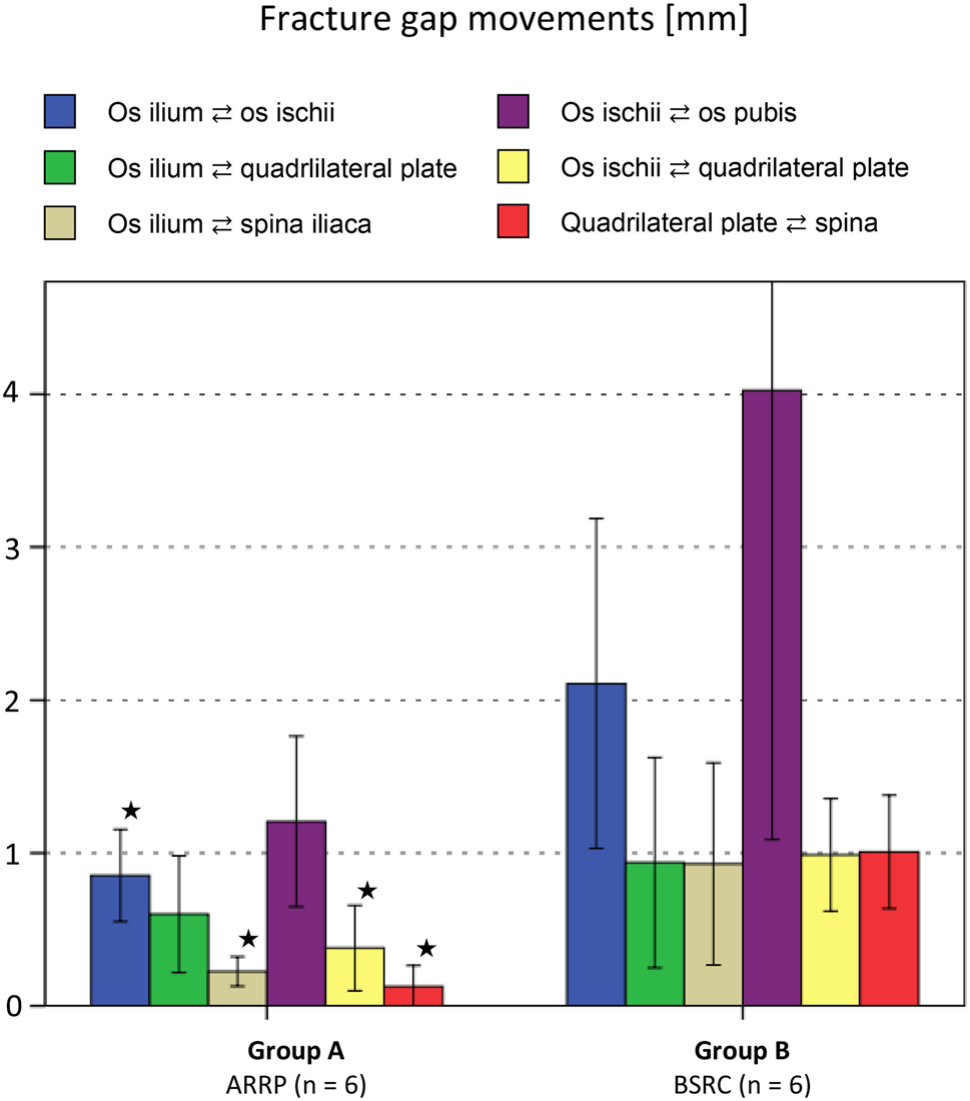
Fracture gap movements for the tested groups A and B (mean value ± 1 SD) after 31,000 cycles under full weight bearing. Student’s *t* tests for independent samples were performed to identify differences in the same fracture gaps among the two groups. Stars indicate statistically significant (*p* < 0.05) results

Fracture gap movements were analyzed after 1000 cycles under partial weight-bearing (250 N) and after 31,000 cycles of loading under full weight-bearing (Figs. 5, 6 and 7). Under partial weight-bearing fracture gap movements were significantly different between the two groups. The higher stability for ARRP was most relevant between the *os ilium* and *os ischii* (44% reduction in movement, *p* = 0.171), and the *os ilium* and *spina iliaca* (57% reduction in movement, *p* = 0.26). In addition, the ARRP group showed significantly less fracture gap movement between the *quadrilateral plate* the *spina iliaca* (91% reduction in movement, *p* = 0.009).

Under full weight-bearing conditions movements between fracture gaps were always larger for the BSRC fixation as compared to the ARRP fixation. The improvements in fixation stability due to the ARRP fixation were most relevant for the *os ilium* to *os ischii* fracture gap (60% reduction in movement, *p* = 0.035) and the *os ilium* and *spina iliaca* fracture gap (76% reduction in movement, *p* = 0.048). ARRP also demonstrated significantly less movement in the fracture gaps *os ischii* to *quadrilateral plate* (62% reduction, *p* = 0.009) and *quadrilateral plate* to *spina iliaca* (87% reduction, *p* < 0.001). Although ARRP fixation showed less motion in the fracture gaps *os ilium* to *quadrilateral plate* (*p* = 0.318) and *os ischii* to *os pubis* (*p* = 0.065), these differences were not statistically significant.

The rotation of the quadrilateral plate was assessed with respect to the *os ilium* (Table 1). After 1.000 cycles of partial weight-bearing fragment rotations around the sagittal axis (*p* = 0.771) was similar for ARRP and BSRC. Nevertheless, the ARRP demonstrated less rotation around the horizontal (*p* = 0.199) and the longitudinal axis (*p* = 0.135). Under full weight-bearing conditions fragment rotation around the sagittal axis was approximately three times larger when a BSRC was used instead of an ARRP (*p* = 0.015). The rotations around the horizontal (*p* = 0.071) and longitudinal axis (*p* = 0.070) tended to be larger for BSRC as compared to ARRP.

**Table 1:** Rotations of the quadrilateral plate fragment with respect to the os ilium after 1000 and 31,000 cycles under partial and maximum load bearing

Rotation (°) (mean ± SD)	(A) ARRP (*n* = 6)	(B) BSRC (*n* = 6)	*p* value
Horizontal axis^a^			
pw	− 0.2 ± 0.8	0.1 ± 0.2	0.199
fw	− 0.2 ± 0.6	2.4 ± 2.8	0.071
Sagittal axis^b^			
pw	− 0.2 ± 0.1	0.2 ± 0.1	0.771
fw	− 1.1 ± 0.9	− 3.0 ± 1.3	0.015*
Longitudinal axis^c^			
pw	− 0.1 ± 0.1	− 0.1 ± 0.1	0.135
fw	− 0.2 ± 0.3	− 1.2 ± 1.0	0.070

*pw* partial weight-bearing, *fw* full weight-bearing

^*^Indicating significant (*p* < 0.05) differences in the Student’s *t* test

^a^Positive sign for anterior superior direction

^b^Positive sign for medial superior direction

^c^Positive sign for medial posterior direction

## Discussion

This biomechanical study compared two different reinforcement techniques for severely displaced acetabular fractures typically seen in geriatric patients. The acetabulum roof-reinforcement plate (ARRP) which employs a special cage fixation at the intact iliac bone in combination with fixed-angle screws demonstrated a consistently improved biomechanical stability compared to the Burch–Schneider reinforcement cage (BSRC) technique. Even under unrestricted partial and full weight-bearing conditions, the ARRP resulted in interfragmentary fracture movements of typically less than 1 mm and can thus provide a biomechanically sound fracture healing environment.

Displaced acetabular fractures with disruption of the quadrilateral plate frequently result in post-traumatic osteoarthrosis and long-term surgery outcome is directly related to accuracy of fracture reduction [23, 27]. Anatomical reduction seems to be essential to achieve a good or an excellent outcome [2, 11, 19, 21, 23, 39]. However, poor bone density as well as the lack of capacity for partial loading increases the risk of secondary joint failure due to loss of reduction. In cases of osteoporotic acetabular fractures in the elderly, surgical fracture fixation alone can yield poor results in patients with several co-morbidities [31]. Several studies have demonstrated that immediate postoperative mobilization with full weight-bearing may be beneficial with regard to avoiding a disease-specific functional outcome [29, 30, 40]. However, the optimal implant configuration has yet to be elucidated.

The indication for primary THA is often discussed and there is no need for primary THA in general cases [35]. However, several studies have shown that, even in patients with an anatomical reduction, a hip joint failure resulting in surgical conversion to total hip replacement within the first 24 months after ORIF occurred in 0–17% of cases [1, 2, 5, 12–14, 16, 18, 20, 32]. In most of these cases, hip joint failure was directly related to displaced acetabular fractures with a dome fragment. However, even in cases without a dome fragment, fracture gap movement of the quadrilateral plate can delay fracture healing considering only partial weight-bearing employed to avoid secondary hip joint failure. Because anatomical fracture gap reduction is not the main target of the ARRP and BSRC, both constructs obtain a congruent hip joint allowing immediate postoperative full weight-bearing mobilization.

In the postoperative simulation starting with partial and ending with full weight-bearing conducted in the present study, the comparison of construct stability among both groups revealed less quadrilateral plate movement for ARRP. Furthermore, the ARRP fixation stability was most significantly relevant under full weight-bearing conditions as compared to BSRC. Thus, protrusion of the femoral head (as indicated by lateral rotation of the quadrilateral plate) was sufficiently prevented by ARRP treated fractures. It is worth noting that testing showed that ARRP distinctly reduced the fracture gap movements in *os ilium* to *os ischii, os ilium* to *spina iliaca, os ischii* to *quadrilateral plate* and *quadrilateral plate* to *spina iliaca*. It was assumed that the cause for higher relative quadrilateral plate and fracture gap movement with BSRC is presumably due to a higher ARRP construct stiffness. The use of 13 angle stable screws fixed in the solid iliac bone seem to provide higher stability compared to the seven non-angle stable screws of the BSRC construct. Angular stability provided by the ARRP screws may be beneficial regarding the prevention of large fracture gap movements. Another reason for the higher stability of the ARRP might be caused by the fact that the monoaxial locking screws with different angles of the individual screws with respect to the implant might provide additional purchase in the iliac bone.

A large movement in *os ischii* to *os pubis* was detected in both groups, although this may not be a disadvantage for both implants and could easily be addressed by methods such as additional plating. However, this would significantly increase operation time and be an unreliable method in geriatric patients favoring immediate postoperative full weight-bearing.

### Limitations

As the model studied represents loading under partial and full weight-bearing conditions, a single-leg stance model was chosen to evaluate fracture gap movement of both implants. In the presented test set-up fracture gap movements in both groups were analyzed at the beginning after 1000 cycles with 250 N and after 31,000 cycles, simulating long-term loading conditions. Egol et al. [8] indicated that secondary screw loosening is sufficiently prevented by the use of locking implants in long-term loading conditions, especially in osteoporotic bones. Secondary screw loosening was unable to be identified in neither the ARRP nor the BSRC group, assuming high stability for both implants. Both constructs require the use of bone cement to fix the cup into the cage, which brings up one limitation for both procedures. Fluid cement running in between the cage and the pelvic bone was observed, which may possibly contribute to higher construct stability.

A cable was used to prevent the bones from collapsing and to achieve a hip joint load as according to Bergmann et al. [3]. High loads led to rope breakage in preliminary tests. Therefore, the maximum axial force of the testing machine had to be limited to 1800 N and the constructs were unable to be loaded until failure. The use of the rope also resulted in a secondary tensile load in the rope due to the primary axial load, which applied additional force to the bones. Since this secondary load was a reactional force that could not be controlled, the overall maximum load fluctuated between 2200 and 2400 N. This difference was classified as negligible, since the data of Bergmann et al. scatter in the same range [3].

### Strengths

This biomechanical study presents several strengths including (1) the use of a clinically relevant acetabular ACPHT fracture model; (2) utilization of fourth-generation Sawbones as a suitable biomechanical comparison for human bone [9, 37]; (3) less interspecies variability of physical properties among specimens due to the use of composite bone models; (4) long-term loading conditions representing a long postoperative phase identifying interspecies differences; (5) implementation of a single-leg stance model demonstrating full weight-bearing conditions.

## Conclusion

In conclusion, this biomechanical study compared two constructs in a clinically relevant scenario for cases of hip joint failure in the elderly with reduced bone quality. No clinically relevant implant failure or loss of reduction was noted in either construct. However, the acetabulum roof-reinforcement plate demonstrated increased fixation stability of the quadrilateral plate under partial and full weight-bearing conditions and provides a possible treatment option for anterior column with posterior hemitransverse acetabular fractures.
