# Correction to: Comparison of two reinforcement rings for primary total hip arthroplasty addressing displaced acetabular fractures: a biomechanical analysis

**DOI:** 10.1007/s00402-020-03475-7

**Published:** 2020-05-27

**Authors:** Johannes Becker, M. Winkler, C. von Rüden, E. Bliven, P. Augat, H. Resch

**Affiliations:** 1 Department of Trauma Surgery, BG Unfallklinik Murnau, Murnau, Germany; 2 Institute for Biomechanics, BG Unfallklinik Murnau, Murnau, Germany; 3 Institute for Biomechanics, Paracelsus Medical University, Salzburg, Austria; 4 Department of Traumatology and Sports Medicine, Paracelsus Medical University, Salzburg, Austria

## Correction to: Archives of Orthopaedic and Trauma Surgery 10.1007/s00402-020-03433-3

The original version of this article unfortunately contained a mistake. Author's surname is written incorrectly “von Rueden” in the original article. “von Rüden” is the correct spelling (also in all his PUBMED articles).

The original article has been corrected.

